# Adolescent Renal Tumours: Diagnostic and Therapeutic Challenges in a Transitional Age Group—A Multidisciplinary Case Report Series from a Single Center

**DOI:** 10.32604/or.2026.072807

**Published:** 2026-03-23

**Authors:** Antonio Ruggiero, Fernando Fuccillo, Valerio Di Paola, Alberto Romano, Palma Maurizi, Dario Talloa, Nazario Foschi, Pierluigi Russo, Marco Racioppi, Stefano Mastrangelo, Giorgio Attinà

**Affiliations:** 1 Pediatric Oncology Unit, Fondazione Policlinico Universitario Agostino Gemelli IRCCS, Rome, Italy; 2 Department of Woman and Child Health and Public Health, Università Cattolica del Sacro Cuore, Rome, Italy; 3 Department of Bioimaging, Radiation Oncology and Hematology, Fondazione Policlinico Universitario A. Gemelli IRCSS, Rome, Italy; 4 Department of Urology, Fondazione Policlinico Universitario Agostino Gemelli, Rome, Italy

**Keywords:** Paediatric oncology, adolescent oncology, renal tumours, Wilms’ tumour (WT), renal cell carcinoma (RCC), case report

## Abstract

**Background:**

The management of renal neoplasms in adolescent patients poses unique clinical challenges due to their transitional position between paediatric and adult populations. This age group exhibits marked heterogeneity in tumour histology, ranging from entities commonly observed in paediatric oncology to tumours typical of adult age, as well as rare histological subtypes that exceptionally affect the kidney. Given the substantial differences in clinical protocols between paediatric and adult populations, rigorous multidisciplinary evaluation is essential to determine optimal diagnostic and therapeutic strategies for adolescent patients.

**Case Description:**

We present four cases from our tertiary referral centre that illustrate the variability in radiological and histopathological presentations and clinical outcomes in this population, underscoring the critical importance of a multidisciplinary approach. Case 1 demonstrates the typical management of Wilms’ tumour in an older paediatric patient. Case 2 exemplifies the diagnostic challenge of distinguishing between Wilms’ tumour and renal cell carcinoma at the upper end of the adolescent spectrum. Case 3 revealed the unexpected diagnosis of renal Ewing sarcoma in a 13-year-old female. Case 4 highlights the potential for severe perioperative complications, including life-threatening thromboembolic events, in a patient with Wilms’ tumour.

**Conclusions:**

The variability in tumour types, biological behaviour, and potential for severe complications underscores the necessity of comprehensive multidisciplinary management in specialized hospital settings. An integrated approach ensures accurate diagnosis, individualized treatment planning, and effective management of complications, ultimately optimizing outcomes for adolescent patients with renal neoplasms.

## 1 Introduction

Epidemiological data from Europe indicate that renal tumours constitute approximately 7% of paediatric malignancies, with Wilms’ tumour (WT) representing the predominant histological subtype (90%). The annual incidence of WT is approximately 1 in 100,000 children, with peak incidence at approximately 3 years of age. Less common paediatric renal tumour histological subtypes include clear cell sarcoma of the kidney (CCSK), malignant rhabdoid tumour of the kidney (MRTK), and renal cell carcinoma (RCC), collectively comprising approximately 6% of childhood renal malignancies. However, by the age of 14 years, RCC represents 50% of paediatric renal tumours [1–4]. In contrast, the histological distribution changes substantially in adults, where renal cancers demonstrate a worldwide annual incidence exceeding 400,000 diagnosed cases, with RCC constituting 80%–90% of all renal malignancies. The predominant subtype is clear cell RCC (70%), while less frequent are papillary (10%–15%) and chromophobe (4%–5%) RCC [5,6]. In Europe, standardized clinical protocols guiding diagnosis and treatment in paediatric and adult patients are defined by the SIOP-RTSG (International Society of Paediatric Oncology—Renal Tumor Study Group) Umbrella Protocol and ESMO (European Society for Medical Oncology) Renal Cell Carcinoma guidelines, respectively. When patients present with symptoms suggestive of renal neoplasia—such as haematuria, flank pain, palpable mass, unexplained weight loss, or hypertension—the initial imaging work-up is similar across age groups. Generally, both cohorts undergo renal ultrasonography, followed by abdominal magnetic resonance imaging (MRI)—or CT scan if MRI is not available—to delineate local tumour extent, and thoracic computed tomography (CT) to evaluate potential metastatic disease plus chest radiography to obtain subsequent reference image for comparison during follow-up [7,8]. According to European data [9,10], the SIOP-RTSG protocol recommends core needle biopsy (CNB) to differentiate between WT and RCC based on specific clinical, radiological and biochemical parameters. CNB is indicated for all children aged 10 years and older and for children between 7 and 10 years of age with tumour volume less than 200 mL. In adolescents above 16 years of age, where RCC is more probable than WT, CNB is not indicated. Beyond these criteria, CNB is reserved for cases of diagnostic uncertainty, such as atypical radiological characteristics, unusual metastatic patterns, and abnormal biochemical findings [7,9,11]. In adults, biopsy is indicated—without clear consensus—for clinical T1a tumours (confined to the kidney, ≤4 cm), given the significant incidence of benign neoplasms in this age group (approximately 30% of renal lesions) [12].

Therapeutic strategies are dictated by accurate staging and histology. In paediatric patients, the SIOP-RTSG approach incorporates preoperative chemotherapy (vincristine plus actinomycin-D) for most WT patients beyond 6 months of age, with surgical timing depending on tumour-related complications such as haemorrhage or rupture. Radical nephroureterectomy (RNU) is the standard for unilateral WT, while nephron sparing surgery (NSS) is reserved for bilateral disease. Risk-adapted postoperative therapy is tailored to pathological findings and staging [7,13,14]. For paediatric RCC, complete surgical resection (R0) is the cornerstone of treatment, typically via RNU, though NSS may be feasible in selected cases [7].

Adult RCC management follows the TNM staging and the IMDC risk stratification (International mRCC Database Consortium) for metastatic disease, incorporating clinical performance status, laboratory abnormalities, and time from diagnosis to systemic therapy [8]. NSS is preferred for T1 tumours (≤7 cm, limited to the kidney) where technically achievable, while RNU is indicated for ≥T2 lesions (≥7 cm, limited to the kidney). NSS is prioritized in patients with solitary kidneys, bilateral tumours, or compromised renal function, regardless of tumour size. Minimally invasive approaches are increasingly adopted, but open surgery remains standard for advanced tumours. Routine adrenalectomy and lymphadenectomy are not recommended without specific indications [15]. In the adjuvant setting, pembrolizumab is currently the only approved therapy, showing improved overall survival (Hazard Ratio 0.62) and disease-free survival (Hazard Ratio 0.72) in the phase III KEYNOTE-564 trial for intermediate- and high-risk post-nephroureterectomy patients [16]. For metastatic RCC, systemic therapy includes PD-1 inhibitors combined with VEGFR-targeted agents (lenvatinib, axitinib, cabozantinib) or CTLA-4 inhibitors (ipilimumab), achieving median OS approaching 4 years [8,17–20]. For patients ineligible for immunotherapy, tyrosine kinase inhibitors remain an option [8,21,22].

This case report series aims to systematically illustrate the diagnostic and therapeutic challenges encountered in adolescent renal tumours through four representative cases from our tertiary centre. Specifically, we sought to demonstrate how tumour histological variability, atypical clinical presentations, and age-specific complications in this transitional population necessitate tailored multidisciplinary approaches that differ from standard paediatric or adult protocols. By presenting diverse clinical scenarios—including diagnostic dilemmas between Wilms’ tumour and renal cell carcinoma, rare histological entities, and severe perioperative complications—we provide evidence-based insights to guide clinical decision-making and optimize therapeutic outcomes for adolescent patients with renal neoplasms.

This study was approved by the Institutional Review Board, Università Cattolica del Sacro Cuore (approval number: DIPUSVSP-09-12-2580). The handwritten informed consent was obtained from the patient. Besides, this study was prepared according to the CARE case report guideline, and a CARE checklist was provided. Please see Supplementary Material S1 for more details.

## 2 Case Series

### 2.1 Case 1

**Figure 1:**
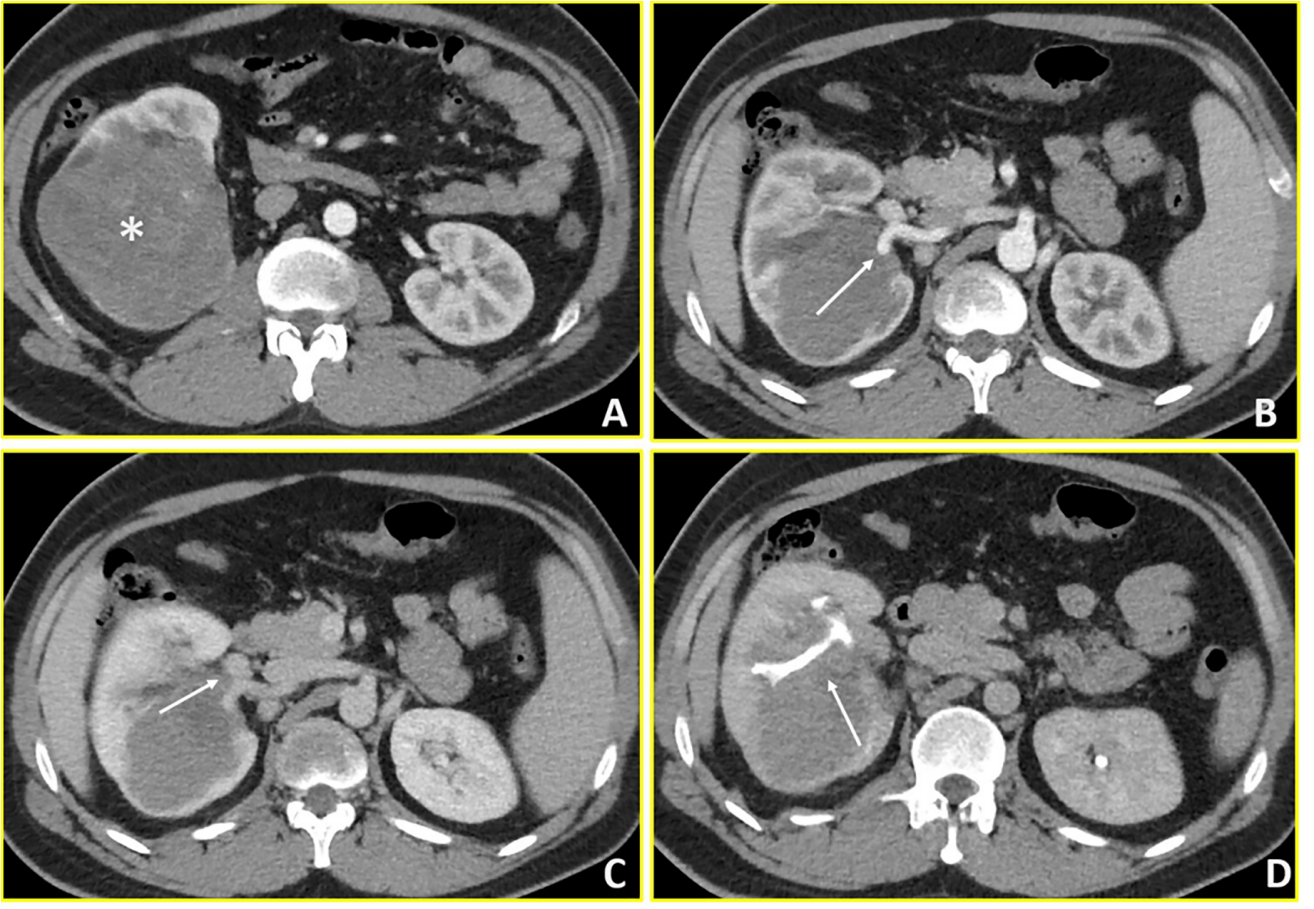
Wilms’ Tumour (Case 1). Contrast-enhanced CT imaging demonstrates a hypovascular mass in the right kidney with extensive vascular and collecting system involvement. (**A**, **B**) Axial arterial-phase images reveal a predominantly hypovascular renal mass (asterisk) with infiltration of the right renal artery (arrow in B). (**C**) Axial venous-phase image shows tumor extension into the right renal vein (arrow). (**D**) Axial urographic-phase image demonstrates infiltration of the renal collecting system with involvement of the renal calyces (arrow).

A 15-year-old male presented to the Emergency Department of Policlinico Gemelli with gross haematuria. Initial abdominal ultrasonography revealed a right renal lesion, subsequently confirmed by contrast-enhanced abdominal CT and MRI as a 13 × 8 × 9 cm neoplasm with infiltration of the renal pelvis, involvement of hilar vessels (both renal artery and vein), and direct contact with the ipsilateral adrenal gland (Fig. 1). Thoracic CT demonstrated no metastatic disease. Following multidisciplinary tumour board discussion (including oncologist, urologist and radiologist), the decision for upfront surgery was made based on the patient aged 15 years and 8 months approaching the 16-year protocol threshold combined with high-risk radiological features: extensive hilar vascular infiltration, intraparenchymal extension with renal pelvis involvement, and immediate risk of complications including haemorrhage or rupture as recognized by SIOP-RTSG guidelines for tumour-related emergencies. The patient underwent immediate video-laparoscopic right nephroureterectomy with adrenalectomy, performed en-bloc to ensure disease-free surgical margins given the intimate tumour-adrenal anatomical relationship demonstrating concern for adherence on imaging. The patient subsequently received post-operative chemotherapy according to the SIOP-RSTG Umbrella protocol. Clinical and radiological surveillance with physical examination, abdominal ultrasound, chest X-ray, urine tests and blood tests were programmed every three months for the first two years, then continued every six months till the fifth year. No disease recurrence was observed after more than three years of follow-up.

### 2.2 Case 2

**Figure 2:**
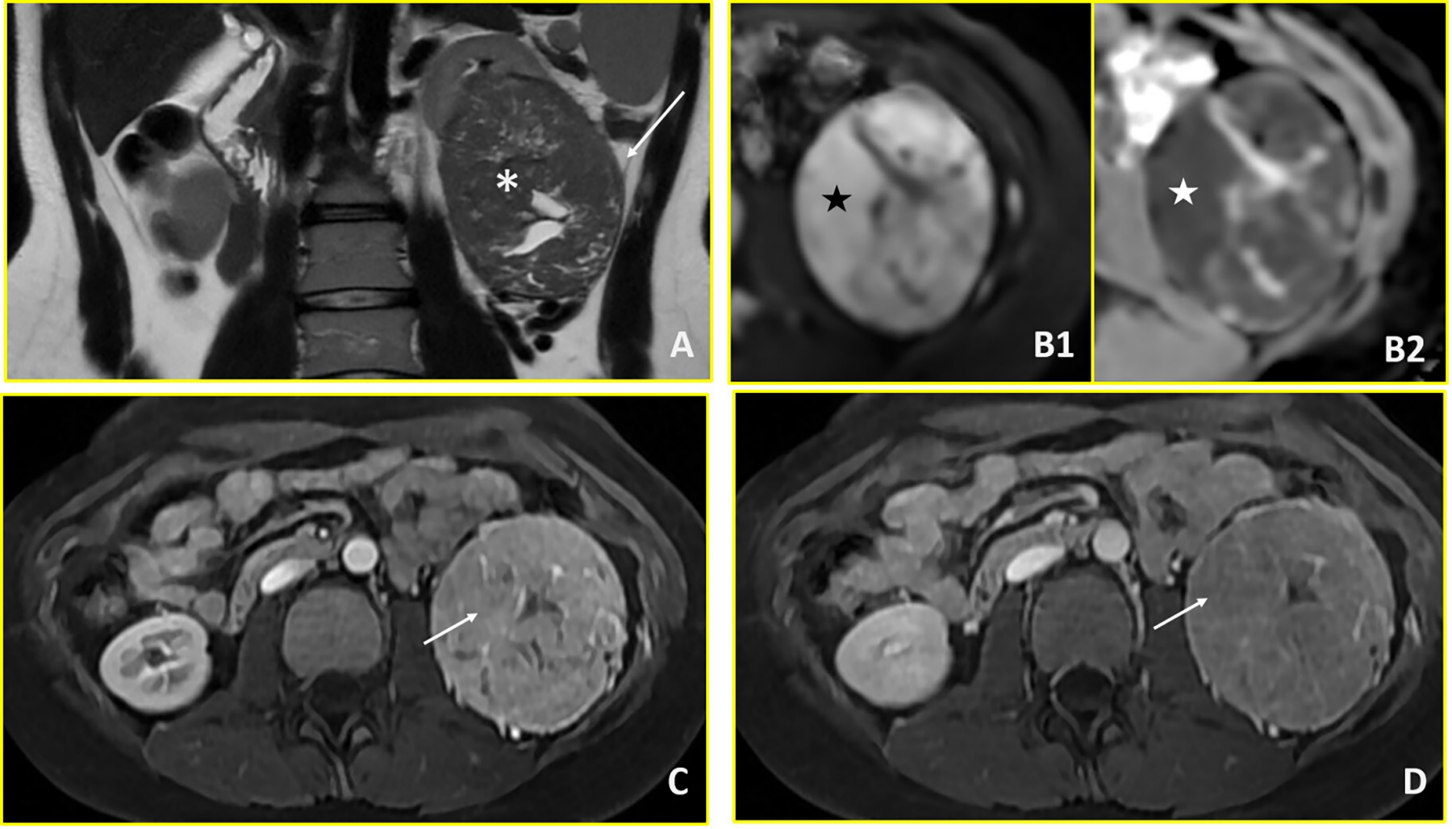
Renal cell carcinoma (Case 2). Multiparametric MRI demonstrates a large left renal mass with imaging features characteristic of renal cell carcinoma. (**A**) T2-weighted axial image shows a hypointense mass in the left kidney (asterisk) with extension into the perirenal fat (arrow). (**B1**, **B2**) Diffusion-weighted imaging reveals restricted diffusion, characterized by hyperintensity on high b-value images (b = 800 s/mm^2^; black star in B1) and corresponding hypointensity on the apparent diffusion coefficient (ADC) map (white star in B2). (**C**, **D**) Dynamic contrast-enhanced imaging demonstrates avid arterial-phase enhancement (arrow in C) with subsequent washout on venous-phase imaging (arrow in D), consistent with the hypervascular pattern typical of renal cell carcinoma.

An 18-year-old female was admitted to the Department of Urology of Policlinico Gemelli with persistent abdominal pain, nausea, and diarrhoea. Initial ultrasonography, followed by abdominal CT, revealed a solid mass of approximately 11 cm at the lower pole of the left kidney, with central necrotic-cystic areas (Fig. 2). Thoracic CT excluded metastatic disease. Further abdominal magnetic MRI provided additional characterization, defining a left renal mass measuring 88 × 75 × 74 mm with well-defined margins and solid composition. MRI signal characteristics showed isointense T1 signal and hypointense T2W signal with hyperintense areas corresponding to fluid/necrotic components. Diffusion-weighted imaging demonstrated restricted diffusion (DWI/ADC). Post-contrast sequences revealed uneven enhancement due to fluid areas without impregnation, with the solid component appearing hypovascular compared to renal parenchyma. Notably, there was no washout in venous and late phases and no evidence of central scar—features that would be expected in typical clear cell RCC. The tumour demonstrated extension into the peri- and sub-renal adipose tissue without signs of extension beyond the retroperitoneum. Importantly, there was no contact with the renal artery or vein. Differential diagnoses included WT and RCC (see Fig. 2). Following discussion between oncologist, radiologist and urologist in a multidisciplinary board review, primary left radical nephroureterectomy was chosen over biopsy, considering the the age of patient. Definitive histopathological examination reported a diagnosis of RCC. Subsequent post-operative imaging excluded residual disease or recurrence. Based on the histological report and the complete removal of the disease, the was no indication for systemic treatment. Subsequently, clinical and instrumental follow-up was initiated, including physical examination, abdominal ultrasound and chest X-ray, as well as urine tests and blood tests including renal function, every three months for the first two years. Subsequent check-ups are expected to take place approximately every six months. Controls will continue for at least five years, considering RCC follow-up protocol.

### 2.3 Case 3

**Figure 3:**
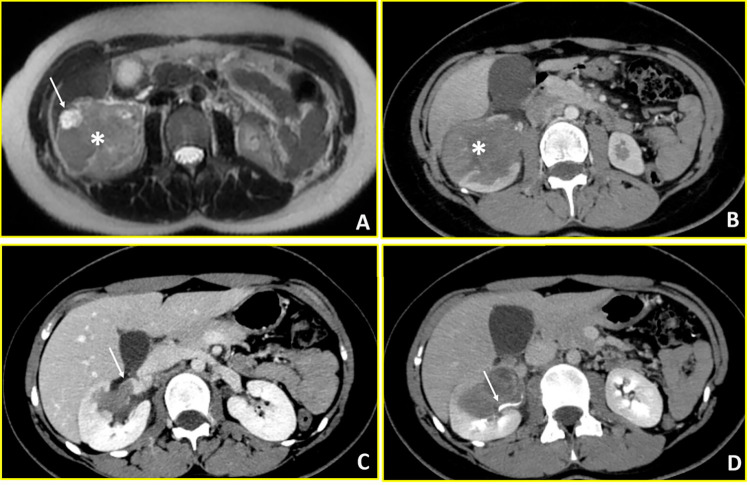
Ewing sarcoma (Case 3). Multimodality imaging demonstrates a large heterogeneous mass in the right kidney with vascular and collecting system involvement. (**A**) T2-weighted axial MR image reveals a heterogeneous renal mass (asterisk) with extensive intralesional necrosis appearing as areas of marked hyperintensity (arrow). (**B**) Axial arterial-phase contrast-enhanced CT image shows a predominantly hypovascular mass (asterisk). (**C**) Axial venous-phase image demonstrates tumor extension into the right renal vein (arrow). (**D**) Axial urographic-phase image shows infiltration of the renal collecting system with involvement of the renal calyces (arrow).

A 13-year-old female presented at the Emergency Department of San Camillo Hospital in Rome with right abdominal and lumbar pain. Abdominal ultrasonography demonstrated a 7.5 cm inferior polar lesion of the right kidney. Abdominal MRI and total body CT confirmed the presence of a voluminous, heterogeneous, expansive lesion affecting the lower middle third of the right kidney with extensive extracapsular involvement at the lower pole, in contact with the ascending colon and the sixth hepatic segment. The tumour architecture was irregularly triangular with the apex extending toward the renal hilum, causing thrombosis of an intrarenal vein branch. Internal heterogeneity was attributed to areas of hemorrhage and necrosis (Fig. 3). The CT scan showed no distant metastases. After these assessments, she was subsequently transferred to Paediatric Oncology Department of Policlinico Gemelli, to complete the diagnostic pathway and define the appropriate treatment. Following multidisciplinary discussion, a percutaneous biopsy was performed, given the patient’s age and atypical radiological features. Initial pathology suggested WT and neoadjuvant treatment was started, using vincristine and actinomycin D for 4 weeks, according to the Umbrella Protocol. However, subsequent molecular analysis identified EWSR1:FLI1 fusion transcript and histological report after complete tumour resection confirmed the diagnosis of Ewing sarcoma. After further collegial discussion involving oncologist and radiotherapist, the treatment was modified to VDC (Vincristine-Doxorubicin-Cyclophosphamide)/IE (Iphosphamide-Etoposide) protocol, including radiotherapy (RT) to the tumour bed (54 Gy on the renal lodge at the surgical site). The patient has recently begun clinical and radiological follow-up, which—given the diagnosis of sarcoma—will include a clinical examination combined with blood tests every three months during the first year, as well as local reassessment with MRI and chest X-ray.

### 2.4 Case 4

**Figure 4:**
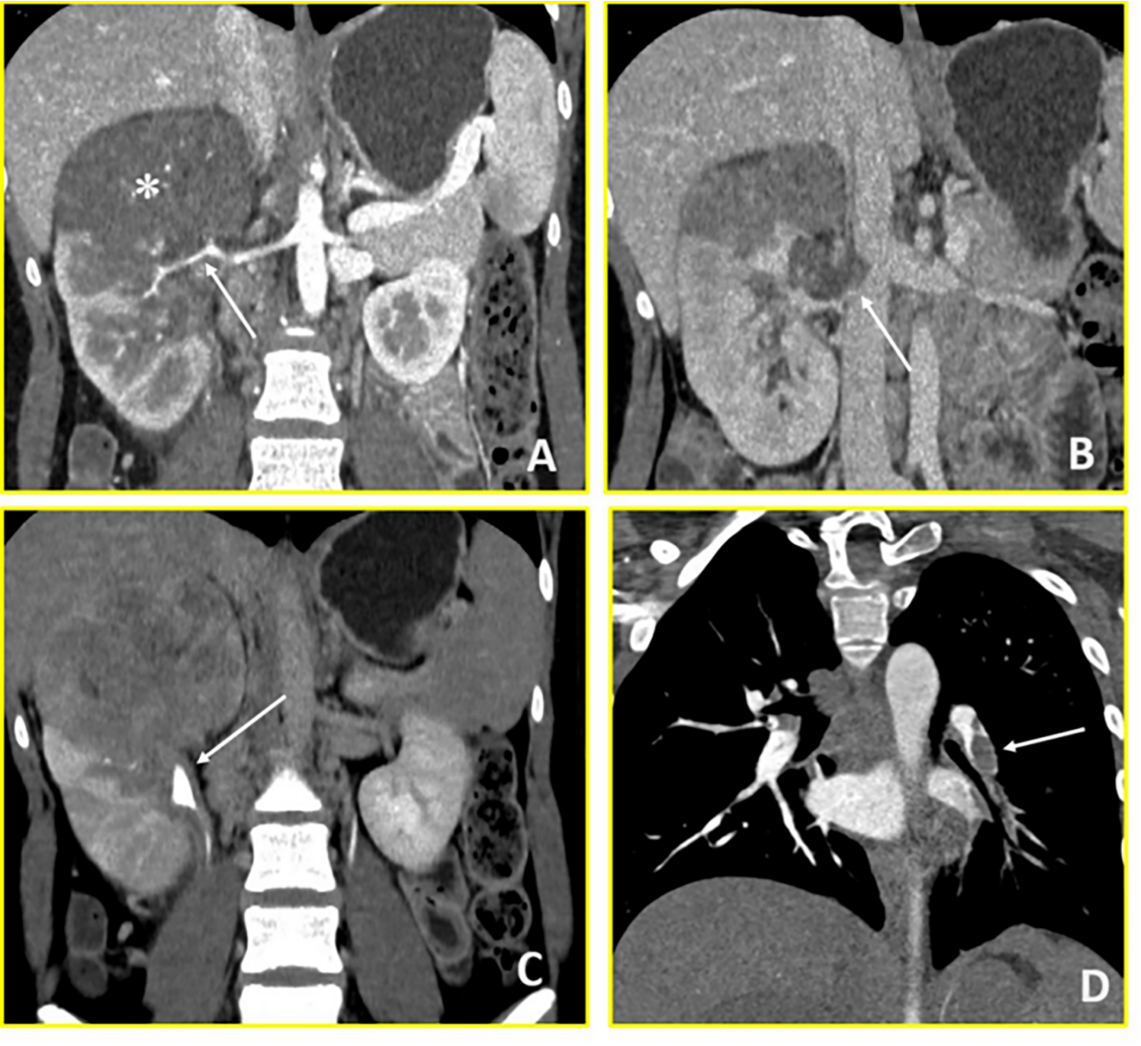
Wilms’ Tumour (Case 4). Contrast-enhanced CT imaging demonstrates a hypovascular right renal mass with vascular invasion and distant thrombotic complications. (**A**) Coronal arterial-phase image reveals a predominantly hypovascular mass in the right kidney (asterisk) with infiltration of the right renal artery (arrow). (**B**) Coronal venous-phase image shows tumor thrombus in the right renal vein with extension into the inferior vena cava (arrow). (**C**) Coronal urographic-phase image demonstrates infiltration of the renal collecting system with involvement of the calyces (arrow). (**D**) Coronal arterial-phase image of the chest reveals a filling defect in the left interlobar pulmonary artery (arrow), consistent with pulmonary thromboembolism.

A 16-year-old female presented to Emergency Department of Policlinico Gemelli with abdominal pain and fever. Thoracic-abdominal CT revealed a large (83 × 80 × 96 mm) inhomogeneous mass at the right kidney’s upper pole with ipsilateral renal pelvis dilatation and right renal vein thrombosis extending into the inferior vena cava. Thoracic imaging scan demonstrated pulmonary thromboembolism with multiple pulmonary infarctions (Fig. 4). The clinical presentation was characterized by recurrent episodes of tachycardia (heart rate > 120 beats per minute). Urgent transesophageal echocardiography was performed, revealing a hyperechoic filamentous formation measuring approximately 2.5 cm, widely mobile from the inferior vena cava, projecting into the right atrium to the tricuspid valve plane. The marked mobility of this atrial thrombus, observed even with patient movements during the examination, indicated extremely high risk of catastrophic embolization or acute right ventricular outflow obstruction. Basal abdominal MRI imaging examination was not possible due to the rapid evolution of the clinical picture. An urgent multidisciplinary discussion involving urologist, cardiac surgeon, anaesthetist, radiologist and oncologist led, due to the condition of haemodynamic instability and the risk of immediate thrombus rupture, to immediate cavectomy, thrombus removal and open right radical nephroureterectomy. Intraoperatively, the patient experienced cardiac arrest requiring cardiopulmonary resuscitation and pharmacological intervention. Post-operative thoracic CT demonstrated massive pulmonary embolism necessitating emergent pulmonary artery thrombectomy. The patient was managed in the Cardiac Intensive Care Unit with continuous intravenous heparin anticoagulation. Once stabilized, she was transferred to the Paediatric Oncology Department on postoperative day one. Histopathological examination with molecular testing confirmed Wilms’ tumour with a biphasic pattern. Following resolution of thromboembolic complications, high-risk post-operatory chemotherapy was initiated according to the SIOP-Umbrella 2016 protocol, alternating Cyclophosphamide/Doxorubicin and Carboplatin/Etoposide courses. Radiotherapy was delivered at week 10 (15 Gy to lungs, 25.2 Gy to tumour bed). End-of-treatment imaging showed complete remission, and the patient remains disease-free at one-year follow-up. In this initial phase of monitoring, her follow-up includes clinical examinations, blood tests, abdominal ultrasound scans and chest X-rays every three months.

A summary of patients’ characteristics is presented in Table 1.

**Table 1:** Summary of clinical cases: demographic, clinical, and diagnostic characteristics.

Case	Age/Sex	Symptoms at Onset	Radiological Findings	Histology	Surgery	Complications	Additional Therapy	Follow-Up
1	15/M	Haematuria	13 × 8 × 9 cm right renal mass with renal pelvis and hilar vessel infiltration	Wilms’ tumour (epithelial predominance), intermediate risk	Video-laparoscopic right nephroureterectomy and adrenalectomy	None	Post-operative chemotherapy per SIOP-RSTG protocol	No recurrence after >3 years
2	18/F	Abdominal pain, nausea, diarrhoea	11 cm left renal lower pole solid mass with necrotic areas	Renal cell carcinoma	Left radical nephroureterectomy	None	Active surveillance with regular follow-up	No recurrence
3	13/F	Right abdominal and lumbar pain	7.5 cm right inferior polar renal lesion with renal vein branch thrombosis	Ewing Sarcoma (EWSR1:FLI1 fusion)	Right radical nephroureterectomy	None	VDC/IE chemotherapy and radiotherapy	Start of regular follow-up
4	16/F	Abdominal pain, fever	83 × 80 × 96 mm right upper pole renal mass with renal vein thrombosis extending to inferior vena cava and right atrium	Wilms’ tumour (biphasic pattern)	Right radical nephroureterectomy, cavectomy and thrombectomy	Intraoperative cardiac arrest, massive pulmonary embolism requiring thrombectomy	High-risk chemotherapy per SIOP-Umbrella 2016 protocol, radiotherapy (lungs: 15 Gy, tumor bed: 25.2 Gy)	No recurrence after 1 year

Note: Abb: VDC/IE, Vincristine-Doxorubicin-Cyclophosphamide/Iphosphamide-Etoposide; SIOP-RTSG, International Society of Paediatric Oncology—Renal Tumor Study Group.

## 3 Discussion

The management of renal neoplasms in adolescents requires rigorous adherence to evidence-based protocols while recognizing that these guidelines must be interpreted within the context of individual clinical presentations. The SIOP-RTSG Umbrella Protocol and COG guidelines provide essential frameworks, but both explicitly acknowledge that complex cases may require individualized decision-making within protocol parameters. The cases presented in this series illustrate both straightforward protocol application and scenarios requiring nuanced interpretation of guidelines, always within a multidisciplinary framework prioritizing patient safety and optimal oncologic outcomes. Case 1 is representative of the typical management of Wilms’ tumour in an older paediatric patient, with uncomplicated surgical intervention followed by standard adjuvant therapy. Case 2 illustrates the diagnostic challenge of differentiating between Wilms’ tumour and RCC at the upper end of the adolescent age spectrum, where histology and therapy diverge sharply. Case 3 highlights the critical role of comprehensive pathological and molecular testing, revealing the unexpected diagnosis of renal Ewing Sarcoma in a 13-year-old female, prompting an entirely different treatment pathway. Case 4 underscores the potential for severe perioperative complications, including life-threatening thromboembolic events, even in histologically typical WT.

Reliance on paediatric algorithms alone may lead to misclassification, therapeutic delay, or inappropriate management, reinforcing the need for comprehensive integration of imaging, histology, and molecular testing supported by multidisciplinary expertise [23]. Treatment planning in adolescents demands an individualized consideration regarding the sequencing of surgery and systemic therapy, guided by a combination of tumour-specific features, patient-related factors, and the collective expertise of all relevant specialists. Advances in surgical technique—including minimally invasive, robotic, and fluorescence-guided resection—offer promise but must be weighed against the risks of rupture, bleeding, or complex vascular involvement [24,25].

In adolescents with renal masses, accurate radiological study and histopathological diagnosis is paramount as therapeutic pathways diverge significantly between WT, RCC and sarcomas. While both WT and RCC affect this age group, their management differs substantially: WT typically require a multimodal approach involving surgery, chemotherapy, and occasionally radiotherapy; conversely, RCC primarily necessitates surgical intervention, with targeted molecular therapies and immunotherapy reserved for metastatic disease [7,8]. The diagnostic complexity is compounded by the fact that the incidence of WT decreases with age, while rarer malignancies such as RCC or sarcomas become proportionally more frequent [4]. To understand these management challenges, it is essential to recognize the unique epidemiological transition that occurs during adolescence. A recent population-based study, considering renal tumours diagnosed between 2001 and 2010 during paediatric age in 308 different registries worldwide, highlighted that renal tumours account for 0.7% of cancers in adolescents across the world. WT represent over 90% of all renal tumours between 1 and 7 years, while the incidence of RCC increases with age becoming the most frequent renal tumour from 14-years patients [4].

The multidisciplinary approach is critical in the diagnostic phase, where the radiologist and pathologist play key roles. In our series, Case 1 exemplifies the nuanced application of protocol guidelines in borderline-age patients with high-risk anatomical features. While the SIOP-RTSG Umbrella Protocol recommends neoadjuvant chemotherapy as standard for Wilms tumour, with biopsy indicated for patients under 16 years with atypical features, the protocol explicitly recognizes that surgical timing depends on tumour-related complications such as haemorrhage or rupture risk [11]. In this patient aged 15 years and 8 months, the multidisciplinary team identified concerning radiological features: extensive hilar vascular infiltration involving both renal artery and vein, intraparenchymal extension with renal pelvis involvement, and tumour proximity to the adrenal gland. The combination of these high-risk features with the patient’s proximity to the 16-year threshold led to the decision for upfront surgery after thorough multidisciplinary discussion. The addition of adrenalectomy was performed to guarantee R0 resection given radiological evidence of tumour-adrenal contact with concern for adherence. While this represents an individualized clinical decision, it remains within the protocol framework that prioritizes patient safety when immediate surgical risks are identified.

Case 2 demonstrates the application of age-appropriate diagnostic algorithms. According to the SIOP-RTSG Umbrella Protocol, biopsy is not indicated for patients aged 16 years and above, given that RCC represents the predominant renal tumour in this age group, accounting for over 50% of renal tumours in adolescents over 14 years [11,26]. At age 18, this patient clearly falls within the age range where adult diagnostic algorithms are applicable. The European Society for Medical Oncology (ESMO) guidelines for RCC management recommend surgical resection as the primary diagnostic and therapeutic approach for localized tumours without metastases [8]. The comprehensive MRI characterization demonstrated imaging features consistent with a solid renal neoplasm with characteristics more suggestive of RCC than WT, including: hypovascular enhancement pattern, absence of classic WT features (such as claw sign or pseudo-capsule), and localized disease without vascular involvement. Given the patient’s age (18 years), imaging characteristics, localized disease and alignment with both SIOP-RTSG age-based recommendations and ESMO adult guidelines, the multidisciplinary team determined that primary surgical approach was appropriate, combining both diagnostic confirmation and definitive treatment in a single procedure.

Case 3 represents a prototypical example demonstrating the critical importance of comprehensive molecular testing in adolescent renal tumours with atypical presentations. Primary renal Ewing sarcoma is exceptionally rare, representing less than 5% of renal tumours and typically affects young adults with a median age around 24 years [27,28]. This tumour lacks pathognomonic radiological features and can easily be confused with other small round blue cell tumours including Wilms tumour, neuroblastoma, rhabdomyosarcoma, and clear cell sarcoma of the kidney.

In our case, cross-sectional imaging demonstrated a large heterogeneous mass with internal haemorrhage and necrosis—characteristics common to several renal tumour types. Given the patient’s age (13 years) and atypical imaging presentation, core needle biopsy was appropriately performed according to SIOP-RTSG Umbrella Protocol recommendations for atypical cases.

The initial histopathological assessment revealed a poorly differentiated malignant neoplasm with high proliferative activity (Ki-67 proliferative index 65%–70%), composed of small-to-medium sized cellular elements with compact chromatin, absent nucleoli, and scarce cytoplasm. Immunohistochemical profiling showed broad negativity for epithelial (AE1/AE3, Cam 5.2), hemato-lymphoid (CD45LCA, CD20, CD3, TdT), and myogenic markers (Desmin, Myogenin, MyoD1), with focal tubular structures positive for epithelial markers arranged in loose stroma. This immunoprofile led to an initial diagnostic impression of Wilms tumour, a well-documented diagnostic pitfall in renal Ewing sarcoma [29].

As described in the literature, the differential diagnosis of small round blue cell tumours of the kidney requires a comprehensive immunohistochemical panel. As recommended by Ellinger et al., essential markers include CD99 (typically positive in Ewing sarcoma with membranous pattern), NSE, vimentin, chromogranin, cytokeratin, and WT-1. However, morphology and immunohistochemistry alone may be insufficient, as CD99 can also be expressed in synovial sarcoma, some Wilms tumours, and lymphoblastic lymphoma [27].

The definitive diagnosis requires molecular confirmation. In over 90% of Ewing sarcoma cases, real time-Polymerase Chain Reaction (real time-PCR) or Fluorescent *In Situ* Hybridization (FISH) demonstrates characteristic translocations, most commonly t(11;22)(q24;q12) resulting in EWSR1:FLI1 fusion. In our patient, molecular testing performed during initial WT-directed neoadjuvant chemotherapy revealed negativity for WT-1, positivity for CD99 staining, and presence of EWSR1:FLI1 fusion protein, establishing the definitive diagnosis of Ewing sarcoma. This prompted immediate therapeutic redirection from SIOP-RTSG WT protocol to Ewing sarcoma-specific VDC/IE chemotherapy plus radiotherapy. This case emphasizes a critical principle: in adolescents presenting with renal masses and discordant clinical-radiological-pathological features, molecular testing is not optional but mandatory to prevent misclassification, inappropriate treatment, and compromised outcomes. The high metastatic potential of renal Ewing sarcoma (53% of cases present with metastases, rising to 59% in adolescents and young adults) and its aggressive nature necessitate prompt and accurate diagnosis [30]. Although our patient initially received WT-directed therapy before molecular confirmation, the rapid identification and treatment modification upon molecular diagnosis ensured appropriate definitive management.

Finally, Case 4 requires detailed discussion given the complexity of managing Wilms tumour with atrial thrombus extension. The SIOP-RTSG protocols strongly advocate neoadjuvant chemotherapy for intravascular thrombus extension, including atrial involvement. This recommendation is supported by substantial evidence. Morris et al. demonstrated, in their analysis of 498 children, that standard-course neoadjuvant chemotherapy (4–6 weeks) achieves thrombus regression in approximately 45%–50% of cases, with significantly better event-free survival (88% vs. 72%) and overall survival (95% vs. 82%) compared to extended courses beyond protocol recommendations [31]. Boam et al. confirmed through systematic review and meta-analysis that neoadjuvant chemotherapy renders approximately 50% of tumour thrombi non-viable, potentially reducing the need for complex vascular surgery including cardiopulmonary bypass [32].

However, these same protocols explicitly recognize specific clinical scenarios requiring immediate surgery. The SIOP approach acknowledges that primary surgery is indicated when the patient demonstrates instability due to thrombus that poses immediate risk of dislodgement causing life-threatening complications [33,34]. In our case, the clinical presentation fulfilled criteria for life-threatening instability: 1. documented hemodynamic instability with recurrent tachycardic episodes (HR > 120 bpm); 2. transesophageal echocardiography demonstrating a large (2.5 cm), widely mobile atrial thrombus with movement extending to the tricuspid valve plane—representing extremely high embolic risk 3. Already-present pulmonary thromboembolism with multiple pulmonary infarctions documented on pre-operative CT 4. Assessment that even minimal delay could result in fatal pulmonary embolism or acute right ventricular outflow obstruction.

Following urgent multidisciplinary discussion involving urologist, cardiac surgeon, anesthesist, radiologist, and oncologist, the decision for immediate surgery was made based on documented life-threatening instability. The surgical approach consisted of: (1) median laparotomy with exposure of the right kidney and inferior vena cava; (2) renal vein incision at its confluence with the inferior vena cava; (3) cranial cavotomy extension for approximately 4 cm to access the intracaval and atrial thrombus; (4) careful thrombus extraction; (5) radical right nephroureterectomy. Intraoperatively, the patient experienced cardiac arrest requiring cardiopulmonary resuscitation and pharmacological support. Post-operative thoracic CT demonstrated massive pulmonary embolism necessitating emergent pulmonary artery thrombectomy via cardiopulmonary bypass with isolation of the left pulmonary artery branch and thrombus removal [33]. It is important to note that the patient had documented pulmonary thromboembolism with multiple infarctions on pre-operative imaging. The post-operative massive embolism likely represented progression of existing disease combined with surgical manipulation of the highly mobile atrial thrombus. While this outcome may appear to contradict the rationale for immediate surgery, the thrombus characteristics (large, mobile, extending to tricuspid valve, already associated with pulmonary emboli) indicated that delaying surgery for even standard-course neoadjuvant chemotherapy carried unacceptable risk of fatal embolization during the treatment window.

Following stabilization in the Cardiac Intensive Care Unit with continuous intravenous heparin anticoagulation, the patient was transferred to Paediatric Oncology and commenced high-risk post-operative chemotherapy (HR1 regimen) according to SIOP-Umbrella 2016 protocol, alternating Cyclophosphamide/Doxorubicin and Carboplatin/Etoposide courses, with radiotherapy delivered at week 10 (15 Gy to lungs, 25.2 Gy to tumour bed). The patient has achieved complete remission with no evidence of disease after one year of follow-up.

This case emphasizes that while neoadjuvant chemotherapy remains the evidence-based standard for Wilms tumour with cavoatrial thrombus, the SIOP protocols appropriately recognize exceptions for patients with life-threatening instability. This case represents such an exception, where documented hemodynamic compromise, extremely mobile atrial thrombus with existing pulmonary emboli, and multidisciplinary assessment of immediate mortality risk necessitated deviation from standard protocol in accordance with established guidelines for life-threatening presentations.

In addition, at the intersection of medical and psychosocial care lies the issue of fertility preservation which should be proactively addressed with adolescent patients and their families prior to treatment initiation, particularly given the potential gonad toxicity of various chemotherapeutic agents. The support of endocrinologist and reproductive medicine expert is integral to supporting the unique needs of adolescents with cancer. All post-pubertal adolescent patients, regardless of sex, facing potential gonadal toxicity should be offered timely counselling and access to established fertility preservation procedures, except for refusal or exceptional reasons forcing the physician to start immediate chemotherapy [35]. The male case of this series (Case 1) accepted cryopreservation by collection of seminal fluid. On the other hand, only two of our female cases, 3 and 4, were offered fertility preservation, since the treatment for Case 2 consisted in surgery only, without any systemic therapy. After deep information about the procedure and its aim, Case 4 refused procedure for personal reasons. Case 3 underwent ovarian tissue cryopreservation, with laparoscopic removal of a fragment of ovarian cortex, freezing and storing in tissue biobank.

An additional, and often underestimated, aspect in the multidisciplinary management of adolescent cancer patients is the lack of integration between paediatric and adult oncology services. The conventional division between paediatric and adult protocols, across nearly all cancer types, restricts clinical trial eligibility for this age group, thereby limiting their access to the full spectrum of available treatments [36]. To address these limitations and ensure optimal care for transitional-age patients, a more unified and collaborative approach is essential. Direct engagement and shared decision-making between paediatric and adult oncologists would further strengthen the decisions of the multidisciplinary team and enhance the delivery of comprehensive care.

Beyond the clinical challenges, psychosocial factors play a critical role in this age group, as cancer diagnosis in adolescence profoundly disrupts a critical developmental stage. During this period, the fundamental psychosocial imperatives of establishing independence, engaging in self-discovery, and fostering meaningful relationships are abruptly side-lined, forcing adolescents to confront existential threats and medical interventions instead of pursuing typical adolescent milestones. Malignancy can have a lasting impact on body image, self-esteem, social relationships, and future aspirations [37,38].

The four cases in our series demonstrate the spectrum of diagnostic and therapeutic challenges in adolescent renal tumours. These cases collectively emphasize that optimal adolescent renal tumour management requires: (1) comprehensive knowledge of evidence-based protocols, (2) recognition of clinical scenarios requiring protocol deviation with appropriate justification, (3) mandatory molecular testing in atypical presentations, (4) multidisciplinary team involvement in complex decision-making and (5) transparent documentation of the rationale for all clinical decisions, particularly when deviating from standard recommendations.

This work presents several limitations, including its design as a single-centre study and retrospective data collection. Furthermore, the small sample size and heterogeneity of histology prevent us from performing a proper statistical analysis reaching more consistent conclusions. Due to the recent start of follow-ups for all patients considered in this study, it is also not possible to definitively describe the long-term outcome in both physical and psychosocial terms.

## 4 Conclusions

This case series highlights the remarkable histopathological diversity and wide range of clinical complexities encountered in adolescent patients with renal tumours. The variability in tumour types, biological behaviour, and potential for severe complications underscores the necessity of comprehensive, multidisciplinary management in advanced hospital settings, equipped with specialized expertise across multiple disciplines. An integrated approach—combining the perspectives of paediatric and adult oncology—ensures accurate diagnosis, individualized treatment planning, and effective complication management, ultimately improving both short- and long-term outcomes. Future research should focus on developing dedicate, evidence-based guidelines specific for the adolescent population, bridging current protocol divisions and enabling access to the full therapeutic spectrum available in both paediatric and adult oncology. These efforts will not only optimize outcomes but also address the unique developmental, psychosocial, and fertility considerations inherent to this age group.

## Supplementary Materials



## Data Availability

The data that support the findings of this study are available from the Corresponding Author, A.R. (Antonio Ruggiero), upon reasonable request.

## Supplementary Materials

The supplementary material is available online at https://www.techscience.com/doi/10.32604/or.2026.072807/s1.

## Abbreviations

Abb: Full term
WT: Wilms’ tumor
CCSK: Clear cell sarcoma of the kidney
MRTK: Malignant rhabdoid tumor of the kidney
RCC: Renal cell carcinoma
SIOP-RTSG: Société internationale d’oncologie pédiatrique-Renal tumor study group
ESMO: European Society for Medical Oncology
MR/MRI: Magnetic resonance/Magnetic resonance imaging
CT: Computed tomography
CNB: Core needle biopsy
RNU: Radical nephroureterectomy
NSS: Nephron sparing surgery
IMDC: International mRCC Database Consortium
Real time-PCR: Real time-Polymerase chain reaction
FISH: (Fluorescent *In Situ* Hybridization)
VDC: Vincristine-doxorubicin-cyclophosphamide
IE: Ifosfamide-Etoposide
