# Supplementary material for: Adolescent Renal Tumours: Diagnostic and Therapeutic Challenges in a Transitional Age Group—A Multidisciplinary Case Report Series from a Single Center
Source: Oncol Res. 2026 Mar 23;34(4):36. doi: 10.32604/or.2026.072807 (PMC13040283; doi:10.32604/or.2026.072807)
Supplement: Supplementary file 1 [file OncolRes-34-72807-s001.docx]

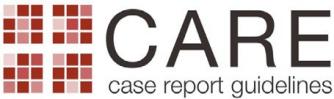

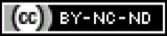

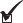
**CARE Checklist of information to include when writing a case report**

| **Topic** | **Item No** | **Checklist item description** | **Reported on Page Number/Line Number** | **Reported on Section/Paragraph** |
| --- | --- | --- | --- | --- |
| Title | 1 | The diagnosis or intervention of primary focus followed by the words “case report” | Page 1/lines 2-3 |  |
| Key Words | 2 | 2 to 5 key words that identify diagnoses or interventions in this case report, including "case report" | Page 1/lines 33-34 |  |
| Abstract  (Structured summary) | 3a | Background: state what is known and unknown; why the case report is unique and what it adds to existing literature. | Page1/lines 26-21 |  |
|  | 3b | Case Description: describe the patient’s demographic details, main symptoms, history, important clinical findings, the main diagnosis, interventions, outcomes and follow-ups. | Page 1/lines 22-28 |  |
|  | 3c | Conclusions: summarize the main take-away lesson, clinical impact and potential implications. | Page 1/lines 29-32 |  |
| Introduction | 4 | One or two paragraphs summarizing why this case is unique **(may include references)** | Page 1-2/lines 84-91 |  |
| Patient Information | 5a | De-identified patient specific information |  | 2 Case series/2.1, 2.2,2.3,2.4 |
|  | 5b | Primary concerns and symptoms of the patient |  | “ |
|  | 5c | Medical, family, and psycho-social history including relevant genetic information |  | “ |
|  | 5d | Relevant past interventions with outcomes |  | “ |
| Clinical Findings | 6 | Describe significant physical examination (PE) and important clinical findings |  | “ |
| Timeline | 7 | Historical and current information from this episode of care organized as a timeline |  | “ |
| Diagnostic Assessment | 8a | Diagnostic testing (such as PE, laboratory testing, imaging, surveys). |  | “ |
|  | 8b | Diagnostic challenges (such as access to testing, financial, or cultural) |  | “ |
|  | 8c | Diagnosis (including other diagnoses considered) |  | “ |
|  | 8d | Prognosis (such as staging in oncology) where applicable |  | “ |
| Therapeutic Intervention | 9a | Types of therapeutic intervention (such as pharmacologic, surgical, preventive, self-care) |  | “ |
|  | 9b | Administration of therapeutic intervention (such as dosage, strength, duration) |  | “ |
|  | 9c | Changes in therapeutic intervention (with rationale) |  | “ |

| Follow-up and Outcomes | 10a | Clinician and patient-assessed outcomes (if available) |  | “ |
| --- | --- | --- | --- | --- |
|  | 10b | Important follow-up diagnostic and other test results |  | “ |
|  | 10c | Intervention adherence and tolerability (How was this assessed?) |  | “ |
|  | 10d | Adverse and unanticipated events |  | “ |
| Discussion | 11a | A scientific discussion of the strengths AND limitations associated with this case report | Page 12/lines 400-404 |  |
|  | 11b | Discussion of the relevant medical literature **with references** | Pages 8-9/lines 220-254 |  |
|  | 11c | The scientific rationale for any conclusions (including assessment of possible causes) | Page 11/lines 379-386 |  |
|  | 11d | The primary “take-away” lessons of this case report (without references) in a one paragraph conclusion | Page 12/lines 393-399 |  |
| Patient Perspective | 12 | The patient should share their perspective in one to two paragraphs on the treatment(s) they received | Pages 12-13/lines 407-416 |  |
| Informed Consent | 13 | Did the patient give informed consent? Please provide if requested | **Yes X** | **No** |

*As the checklist was provided upon initial submission, the page number/line number reported may be changed due to copyediting and may not be referable in the published version. In this case, the section/paragraph may be used as an alternative reference.
